# Metabolic Dependencies in Pancreatic Cancer

**DOI:** 10.3389/fonc.2018.00617

**Published:** 2018-12-12

**Authors:** Ali Vaziri-Gohar, Mahsa Zarei, Jonathan R. Brody, Jordan M. Winter

**Affiliations:** ^1^School of Medicine, Case Western Reserve University, Cleveland, OH, United States; ^2^Department of Veterinary Physiology and Pharmacology, Texas A&M University, College Station, TX, United States; ^3^Department of Medicine, Brigham and Women's Hospital and Harvard Medical School, Boston, MA, United States; ^4^Division of Surgical Research, Department of Surgery, Jefferson Pancreas, Biliary and Related Cancer Center, Jefferson Medical College, Thomas Jefferson University, Philadelphia, PA, United States; ^5^Department of Surgery and Division of Surgical Oncology, University Hospitals Cleveland Medical Center, Cleveland, OH, United States

**Keywords:** pancreatic cancer, metabolism, redox homeostasis, metabolic dependencies, targeting metabolism

## Abstract

Pancreatic ductal adenocarcinoma (PDA) is a highly lethal cancer with a long-term survival rate under 10%. Available cytotoxic chemotherapies have significant side effects, and only marginal therapeutic efficacy. FDA approved drugs currently used against PDA target DNA metabolism and DNA integrity. However, alternative metabolic targets beyond DNA may prove to be much more effective. PDA cells are forced to live within a particularly severe microenvironment characterized by relative hypovascularity, hypoxia, and nutrient deprivation. Thus, PDA cells must possess biochemical flexibility in order to adapt to austere conditions. A better understanding of the metabolic dependencies required by PDA to survive and thrive within a harsh metabolic milieu could reveal specific metabolic vulnerabilities. These molecular requirements can then be targeted therapeutically, and would likely be associated with a clinically significant therapeutic window since the normal tissue is so well-perfused with an abundant nutrient supply. Recent work has uncovered a number of promising therapeutic targets in the metabolic domain, and clinicians are already translating some of these discoveries to the clinic. In this review, we highlight mitochondria metabolism, non-canonical nutrient acquisition pathways (macropinocytosis and use of pancreatic stellate cell-derived alanine), and redox homeostasis as compelling therapeutic opportunities in the metabolic domain.

## Overview of Pancreatic Cancer Treatment and Biology

Pancreatic cancer is the third leading cause of cancer-related death in the United States (1). The disease is predicted to be the second leading cause within the next decade (2). Cures are exceedingly rare, and the 5-years survival for patients with metastatic disease is just 3%. Even patients with localized PDA who undergo resection with curative intent have a 5-years survival of only 30% (1). This survival rate is by far the lowest of the common cancers, and is attributable in large part to PDAs uniquely aggressive behavior and resistance to conventional therapy (3, 4).

The majority of pancreatic cancer patients already have experienced macroscopic or microscopic spread at the time of diagnosis (5–7). Cytotoxic chemotherapeutic agents are the only approved systemic treatments for these patients, and are grouped into two separate multi-agent regimens used as standard-of-care: (1) gemcitabine and nab-paclitaxel (albumin-bound paclitaxel or Abraxane) or (2) 5-fluorouracil, leucovorin, irinotecan, and oxaliplatin (FOLFIRINOX). Unless researchers discover effective strategies to detect PDA earlier or preventative tactics, new ways to treat invasive PDA are desperately needed (8, 9).

Pancreatic cancer develops over many years due to the additive effects of numerous genetic changes (10). Gain-of-function mutations in KRAS at codons 12, 13, and 61 are observed in over 90% of PDAs. Loss-of-function mutations in three specific tumor suppressor genes occur in the majority of PDAs: TP53, CDKN2A, and SMAD4 (11–14). These oncogenic changes occur early in the adenoma to carcinoma progression (15, 16). The vast majority of pancreatic cancers are sporadic. Approximately 10% of patients have one or more immediate family members with a history of PDA. Germline culprits include genes that are important for DNA repair, such as BRCA2, PALB2, ATM, FANCC, and FANCG genes (11, 17).

Histologically, pancreatic tumorigenesis passes through three non-invasive, pre-malignant stages before acquiring an invasive phenotype. The premalignant stages are referred to as pancreatic intraepithelial neoplasia (PanIN 1, 2, and 3). More detailed descriptions of the genetics and pathobiology of pancreatic cancer appear elsewhere (18–20). In addition to well-characterized genetic mutations, there other molecular abnormalities common to PDA including hyperactivated growth factor signaling, epigenetic changes, dysregulated gene expression (transcriptional or post-transcriptional), and abnormal post-translational modifications (18, 21–23).

## The Tumor Microenvironment and Implications for an Aggressive PDA Phenotype

The PDA tumor microenvironment is characterized by one of the most abundant stromal compartments of any tumor type, and this feature is the principal biologic driver of the PDA metabolic program. Neoplastic epithelial cells account for roughly 10–15% of the tumor mass, while non-neoplastic elements comprise the remainder of the tumor. The stroma, or desmoplastic response, consists of an extracellular matrix and diverse cellular elements including fibroblasts, myofibroblasts, lymphatic vessels, blood vessels, pancreatic stellate cells, and immune cells (24, 25). As a result of these elements, the stroma is extremely dense, with a high interstitial fluid pressure (26). Consequently, blood vessels are compressed by biochemical forces and microscopic arteriolar-venular shunts are common events (27, 28). Compounding this significant biologically relevant perfusion challenge, microscopic vessel density is markedly reduced in PDA stroma as compared to normal pancreata (26).

**Figure 1:**
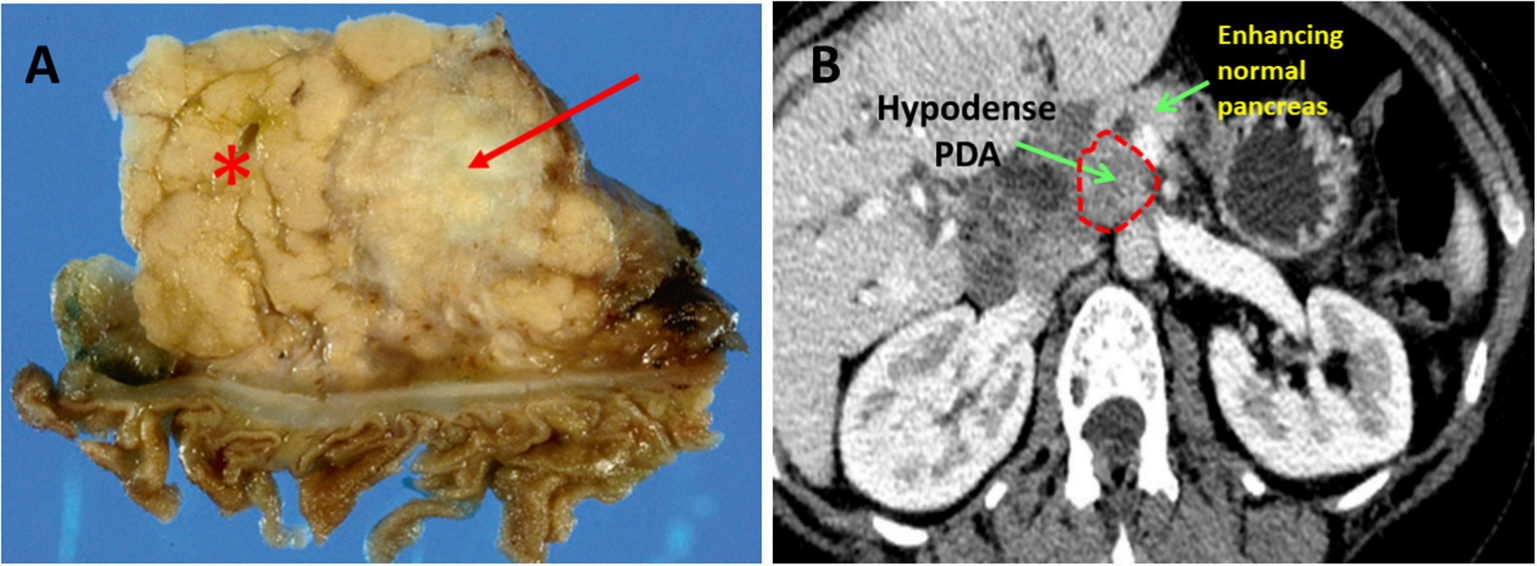
Pancreatic ductal adenocarcinoma. **(A)** Resected human PDA. The arrow identifies the characteristically pale, gray, and hypovascular pancreatic ductal adenocarcinoma. The asterisk marks pancreatic parenchyma. **(B)** On CT scan imaging with intravenous contrast, PDA appears hypodense (dark gray), while well-perfused normal pancreatic parenchyma shows bright enhancement due to penetration by the contrast.

PDA hypovascularity is easy to appreciate macroscopically. Transected PDAs are pale gray and necrotic (Figure 1A). On imaging, PDA appears as a hypodense tumor, easily distinguished by the well-perfused and contrast-enhancing normal pancreatic parenchyma (Figure 1B). This biologic reality accounts for the harsh, nutrient deprived, and hypoxic conditions that are hallmarks of the PDA microenvironment. Indeed, PDA survives, and thrives, in a desert!

Studies implicate mutant KRAS as a key driver of the desmoplastic response. Temporally, this genetic model fits since KRAS mutations arise in PanIN1 lesions when the stroma first develops. As evidence, withdrawal of mutant KRAS expression using shRNAs in genetically engineered KPC mice (autochthonous PDA mice with conditional expression of oncogenic KRAS and TP53 mutations) (16) resulted in the disappearance of the stromal compartment (29).

## Glucose Metabolism

Oxidative phosphorylation is an energy extracting process where pyruvate enters the mitochondria matrix via pyruvate translocase and is oxidized to generate ATP, H_2_O, and CO_2_. Oxygen acts as a final electron acceptor in the electron transport chain. As a result of the associated redox reactions, an electrochemical potential is generated across the inner mitochondrial membrane, which drives a proton-motive force across the membrane, and directly results in the formation of ATP. Thus, carbon flows through the tricarboxylic acid (TCA) cycle and is oxidized to its simplest form, creating basic energy subunits with high energy phosphate bonds that drive other chemical reactions required for cell viability. Well-perfused and differentiated normal cells generate the bulk of their cellular energy through oxidative phosphorylation in the mitochondria (30). Cancer cells, on the other hand, are often oxygen-poor. This scenario theoretically poses an obstacle for effective oxidative phosphorylation. Even in the presence of sufficient oxygen, however, scientists believed for decades that cancer cells were reprogrammed.

Scientists posited that cancer cells relied on cytosolic glycolysis to produce ATP instead of oxidative phosphorylation, even though the energy yield was far less efficient (2 ATP vs. 36 ATP). A preference for aerobic glycolysis is eponymously referred to as the “Warburg's effect” (31), and there have been numerous lines of evidence that in fact reveal robust glycolytic activity in pancreatic cancer cells in certain experimental models, including patient samples. For instance, endogenous expression of many glycolytic enzymes is increased, including hexokinase 2, enolase 2, and lactate dehydrogenases (both LDHA and LDHB isoforms) (32–35). Consequently, glycolytic metabolites, including lactate, are also elevated in pancreatic cancer cells (32, 33, 36).

Generally speaking, a biochemical penchant for glycolysis can serve cancer cells in multiple ways. First, Lactic acid build-up reduces cellular and extracellular pH. This contributes to invasiveness by promoting genetic changes in PDA cells (spurring on genetic selection), impairing the anti-tumor immune response (protecting cancer cells from immune patrol), reducing adherens junctions on cancer cell membranes (facilitating detachment and metastases), and hydrolysis of extracellular proteins to encourage cell invasion (37). Just as important, enhanced glycolytic activity minimizes combustion of carbon from glucose to CO_2_. Instead, organic carbon is preserved, and diverted into cellular building blocks through biosynthetic pathways, such as lipid synthesis, the hexosamine biosynthesis pathway, and the pentose phosphate pathway. This reprogramming effort promotes the macromolecular synthesis needed for cell proliferation (i.e., anabolism) (30, 38, 39). As stated above for desmoplasia (and a common theme for many of the adaptive reprogramming responses described below), oncogenic KRAS drives glycolytic activity in PDA. For instance, KRAS activation leads to increased glucose uptake and elevated levels of glycolysis-associated metabolites (40, 41). *In vivo* models modified by an inducible KRAS extinction mechanism in pancreatic tumors corroborate these findings (29).

Additional studies show that oncogenic KRAS activity supports glycolysis by replenishing the supply of NAD^+^, via upregulation of NAD(P)H oxidase (42). At a regulatory level, the Pasteur effect (an increase in glucose consumption and lactic acid fermentation under low oxygen conditions) is also encouraged by certain transcription factors and related proteins (34, 43). As examples, HIF-1α and MUC1 upregulate glucose transporter 1 (GLUT1) and aldolase A, which leads to increased glucose uptake and glycolysis. MUC1 collaborates with HIF-1α for this purpose. Additionally, under hypoxic conditions, pyruvate dehydrogenase kinase 1 (PDHK1) protein expression is increased, leading to a reduction in pyruvate dehydrogenase (32, 44, 45) activity, and consequently a reduction in oxidative phosphorylation (46).

## The Importance of Mitochondrial Function in PDA

As it turns out, the classic Warburg model misses a large part of the story. Current perspectives maintain that a balance between aerobic glycolysis and oxidative phosphorylation is much more complex, and seems to be highly variable between different tumor types. Moreover, the balance is not fixed in a given tumor. Rather, the metabolic program is dynamic, and responds to ambient conditions (47). Contrary to Warburg's teachings, mitochondria in cancer are often highly functional, and aerobic respiration is even critical for cancer cell survival (48). A preponderance of new evidence shows that pancreatic cancer cells are especially dependent on mitochondrial oxidative phosphorylation under low nutrient conditions, and that mitochondrial metabolism represents a key metabolic vulnerability (47–51).

Diverse macromolecular substrates are utilized by pancreatic cancer cells for catabolic and anabolic purposes, but glucose is consistently viewed as the most important nutrient. Glucose is even likely more limiting in the microenvironment than oxygen, especially in poorly perfused PDA. Oxygen levels exist in the microenvironment around 1.5% (compared to 21% in the atmosphere and 5% in normal tissues (52). At these low levels, mitochondria still function relatively well. In fact, mitochondria perform sufficiently at oxygen levels as low as 0.5% (53, 54). Glucose concentrations frequently dip below 1 mM in poorly perfused tumors, and these levels are profoundly deleterious. Cell necrosis is the outcome at these levels, even when sufficient oxygen is present (55).

**Figure 2:**
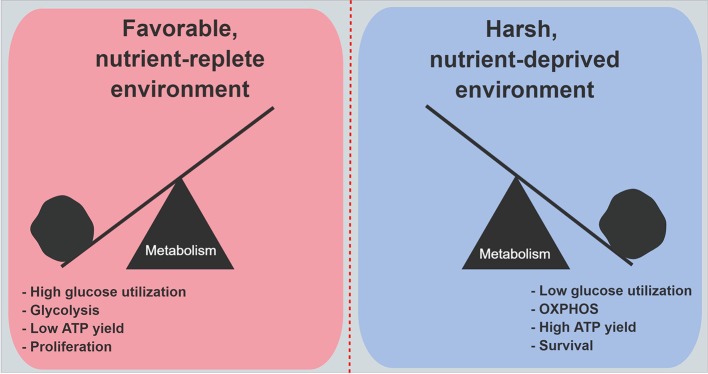
Metabolic features in PDA cells under nutrient abundance and deprivation. Under nutrient abundance, PDA cells have a proliferative phenotype and macromolecular synthesis is prioritized over ATP generation. Under nutrient deprivation, PDA cells have a survival phenotype, and nutrient conservation with maximal ATP generation are prioritized. OXPHOS, oxidative phosphorylation.

An siRNA screen of 2,752 metabolic genes revealed that mitochondrial genes encoding electron transport chain components were functionally the most important genes in cancer cell survival under low glucose conditions (51). Moreover, when 28 cell lines were evaluated for their ability to withstand low glucose conditions, resistant cell lines consistently increased oxygen consumption on demand, as compared to the most vulnerable cell lines. Further, the vulnerable cell lines exhibited higher rates of genetic mutations in mitochondria-encoded electron transport genes (51). Studies revealed that under low glucose, cell proliferation markedly decreases (49, 56, 57). All of these findings suggest a model where cancer cells that are well-adapted to harsh and unfavorable metabolic conditions prioritize the conservation of glucose, and reprogram their biology to maximize energy yields through enhanced mitochondrial respiration. This metabolic shift ensures the production of sufficient ATP to power critical cellular processes. Under austere conditions, cancer cells choose to divert carbon away from biosynthetic pathways, which minimizes unnecessary ATP utilization. Cell proliferation is deferred for a later time when energy supplies become more available, or the cells become even more adept at scavenging nutrients from a deprived microenvironment (Figure 2).

A number of bioenergetic observations support this model. As glucose levels decline, cellular respiration markedly increases. The mitochondrial matrix becomes more acidic as protons return to the matrix through ATP synthase, and hydroxide anions are expelled in phosphate/OH^−^ exchangers. This supports ATP production. A burst of oxidative activity is usually observed (49). Morphologically, the matrix condenses, and cristae expand in functional and working mitochondria. Mitochondrial fusion and elongation are favored over fission events (58–60). Inhibition of an important fission-related protein, dynamin-related protein 1 (Drp1), has been shown to shift the balance from mitochondrial fission to fusion under low nutrient conditions (61).

We have recently found that an RNA-binding protein and regulator of acute survival processes, HuR (ELAVL1), plays an important role in increasing mitochondrial performance in stressed PDA cells. This occurs over a very short timescale. We observed that HuR-proficient PDA cells cultured under low glucose conditions exhibited higher rates of oxygen consumption and ATP production, as compared to isogenic HuR-deficient cancer cells (50). Additionally, HuR expression promoted mitochondrial biogenesis (Vaziri-Gohar, unpublished). Consequently, HuR-deficient PDA cells were unable to ramp up mitochondrial activity under metabolic stress, and were especially vulnerable under low glucose conditions *in vitro*.

Thus, it stands to reason that mitochondrial biology represents a promising therapeutic target in nutrient-deprived cancers (e.g., PDA). There are some clinical studies that support this idea in pancreatic cancer patients. A common diabetic drug, metformin, inhibits complex 1 of the electron transport chain. A meta-analysis of nine retrospective studies and two randomized studies in patients with pancreatic cancer revealed that metformin use was associated with prolonged survival (62–64). Notably, however, the two randomized, phase II studies included in the meta-analysis (both in patients with advanced PDA) were negative studies (63, 64). Prolonged survival was only observed in patients with localized PDA, suggesting that the drug may not be effective in patients with macroscopic distant disease (62). The strategy may be correct, but for improved efficacy, a more potent drug may be required.

More recently, a novel mitochondrial inhibitor was tested in a phase I study of patients with advanced pancreatic cancer. The results were extremely promising. CPI-613 is a lipoic acid analog that disrupts the activity of two mitochondrial enzymes: pyruvate dehydrogenase and α-ketoglutarate dehydrogenase (65, 66). The drug was given to 18 patients at a maximum tolerated dose of 500 mg/m^2^ on days 1 and 3, of a 2-weeks cycle, and in combination with modified FOLFIRINOX (67). The disease control rate, response rate, and complete response rate were 89, 61, and 17%, respectively. In contrast, the rates for FOLFIRINOX alone in a prior phase III study are 71, 32, and 0.6%, respectively (68). A registration phase III trial is planned to begin shortly as of this manuscript writing, and we are poised to test the same combination in a phase II trial of patients with locally advanced PDA at our institution. These studies are expected to be actively accruing by the start of 2019.

## Nutrient Acquisition Pathways

PDA cells also adapt to low nutrient conditions by recruiting unconventional nutrient sources or biochemical salvage pathways to meet bioenergetic demands in a nutrient-deprived microenvironment. There is a small, but important body of literature that highlights some of these biologic processes. Like mitochondrial biology, these non-canonical metabolic pathways represent novel therapeutic opportunities. We have categorized them as intracellular processes (*intrinsic*) or those dependent on the microenvironment (*extrinsic*). While some salvage pathways, like nucleotide salvage, are not directly addressed here, we have focused on ones that have received attention in the recent pancreatic cancer literature.

## Intrinsic Nutrient Acquisition Mechanisms

### Autophagy

Autophagy is a cytoplasmic recycling process that breaks down dysfunctional organelles and unfolded proteins into their basic components for reuse by cells. Mechanistically, unwanted structures are enwrapped by a double-membrane structure called a phagophore to produce an autophagosome. When the vesicle fuses with a lysosome, the contents are enzymatically digested. Autophagy is driven by starvation-induced activation of AMPK, and is suppressed by mTORC1 in normal cells (69, 70). However, oncogenic KRAS signaling appears to regulate or induce autophagy in PDA cells (71, 72). Conceptually, autophagy functions as an effective nutrient salvage pathway for KRAS-driven PDA, especially when extrinsic nutrient sources are deficient. Cleaved LC3 is an indicator of active autophagy (70, 73) and is increased in late PanIN lesions, as well as in PDA (74). Increased autophagy markers have also been associated with worse prognosis in patients with PDA (75). At the subcellular level, inhibiting autophagy in PDA cells disrupts mitochondrial oxidative phosphorylation and exacerbates oxidative damage (74). Autophagy inhibitors like chloroquine and Bafilomycin A1 have been shown to reduce PDA growth in cell culture and pre-clinical animal models (74, 76).

### NAD^+^ Salvage Pathway

NADH is an essential cofactor for enzymatic reactions in both glycolysis and respiration. The molecule provides reducing equivalents for the electron transport chain, which drives the proton-motive force to ultimately yield ATP for basic cellular functions. The regeneration of NAD^+^ as an upstream substrate of NADH production is, therefore, an absolute requirement PDA cell survival, particularly when mitochondrial demands escalate. Tryptophan is the principal source of NAD^+^ production through canonical biochemical pathways (77). However, PDA cells show increased reliance on a separate NAD^+^ salvage pathway. In the cancer-associated salvage pathway, nicotinamide phosphoribosyltransferase (NAMPT) is the rate-limiting step in the production of the NAD^+^ precursor molecule nicotinamide mononucleotide (NMN). NAMPT expression was found to be elevated in PDA cell lines and tissues (78), and its expression was inversely linked to miR-206 activity (78). Based on these findings, NAMPT is recognized as another promising metabolic target against PDA. Inhibitors like FK866 and STF-118804 reduce NAD^+^ levels, glycolytic activity, and mitochondrial function. Importantly, these drugs reduced PDA growth both *in vitro* and *in vivo* (78–80).

## Extrinsic Nutrient Acquisition Mechanisms

When glucose is scarce, amino acids are able to fuel the tricarboxylic acid cycle through various anaplerotic reactions that involve the conversion of aspartate to oxaloacetate (aspartate transaminase), glutamate to α-ketoglutarate (glutamate dehydrogenase), or alanine to pyruvate (alanine transaminase). Biologic processes exploited by PDA cells to extract these anaplerotic substrates from the microenvironment represent additional metabolic dependencies (81), and consequently are also metabolic vulnerabilities and therapeutic targets in the context of an austere PDA microenvironment.

### Macropinocytosis

As with so many other adaptive responses used by PDA cells in the context of severe stress, macropinocytosis is enhanced by oncogenic KRAS (35, 82). In this cellular process, the plasma membrane envelops ambient polypeptides, such as albumin within a macropinosome. Like autophagy, the protein containing vesicle fuses with lysosomes, and proteins are proteolyzed into constituent amino acids (83). Glutamine is the most abundant amino acid released through this process. As a result of macropinocytosis, PDA cells are able to sustain the tricarboxylic acid cycle in the absence of glucose or free glutamine. Targeted inhibition of macropinocytosis with 5-(*N*-ethyl-*N*-isopropyl)amiloride (EIPA) in tissue culture models impaired PDA growth (35). *In vitro* studies revealed that PDA cells experienced sustained viability in the absence of essential amino acids, as long as albumin was present in the media (84). When resected PDA tissues were incubated with tetramethylrhodamine-conjugated dextran (TMR-dextran) for detection purposes, macropinosomes were clearly visualized by immunofluorescence microscopy (84).

### Pancreatic Stellate-Derived Alanine

While PDA stroma has relatively low levels of free nutrients, the stroma is, in fact, replete with non-neoplastic cell types that are potential fuel sources. Pancreatic stellate cells appear important to PDA cells toward this end. These cells are myofibroblasts that generate the extracellular matrix in the exocrine pancreas and provide a scaffold for desmoplasia in PDA. Studies indicate that pancreatic stellate cells produce alanine by autophagy. Interestingly, PDA cells appear to stimulate this process, possibly through paracrine signaling, although the inducing factor remains unknown. Free alanine excreted by pancreatic stellate cells is imported into PDA cells and converted into pyruvate by alanine transaminase. Alanine anaplerosis than supplies the tricarboxylic acid cycle to sustain it under nutrient stress (85). The authors refer to the communication and interdependence between PDA cells and pancreatic stellate cells as an example of metabolic cross-talk. It is becoming increasingly clear that multi-lineage 3-D models to study metabolic cross-talk in PDA may yield new key insights into additional metabolic vulnerabilities.

**Figure 3:**
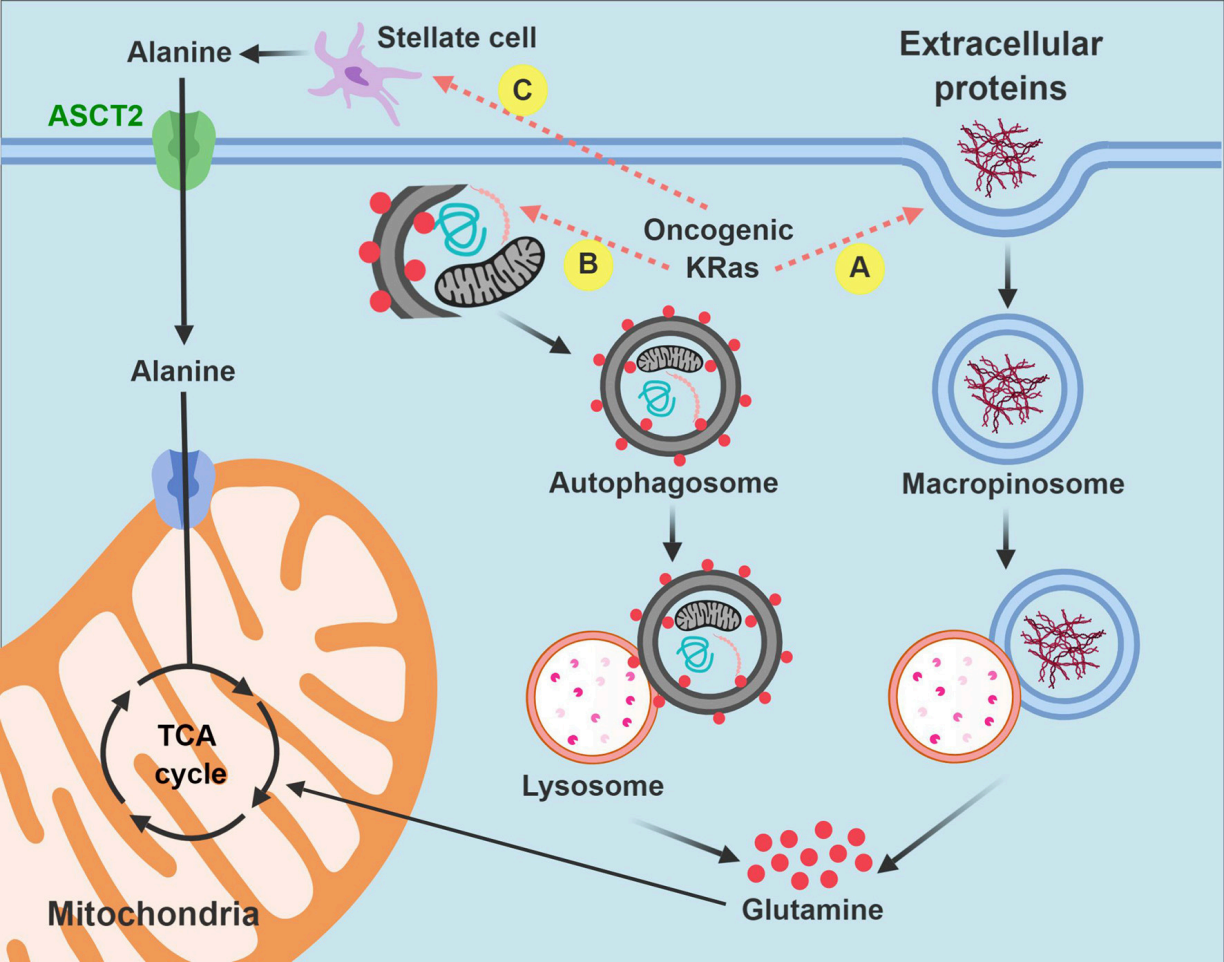
Intrinsic and extrinsic nutrient acquisition pathways in PDA cells. PDA cells hijack stromal elements to fuel the tricarboxylic acid (anaplerosis) and other biochemical processes when nutrients are limited. Carbon is extracted by macropinocytosis **(A)** and autophagy **(B)** after auto-digestion by lysosomes. PDA cells also stimulate pancreatic stellate cells to produce and excrete free alanine **(C)**. Non-functional mitochondria are dark in the figure and are targeted for autophagy. Functional mitochondria are colored pink. TCA, tricarboxylic acid.

In summary, macropinocytosis and metabolic cross-talk between PDA cells and pancreatic stellate cells offer two compelling examples of how PDA cells hijack existing biologic processes or resources from the microenvironment for their own survival advantage. By recruiting these pathways, PDA cells have discovered alternative strategies to fuel the tricarboxylic acid cycle and meet their bioenergetic when the pantry is otherwise bare (Figure 3).

### Redox Homeostasis

PDA cells utilize a positive feedback loop between oncogenic KRAS signaling and reactive oxygen species (ROS) to sustain tumor growth (86, 87). This interplay seems to be especially important early in the adenoma-to-carcinoma progression sequence when ROS levels are manageable. ROS stimulates other pro-growth pathways early on in cancer progression as well, like PI3K signaling (88). The generation of genetic mutations by ROS may also play a tumor-promoting role in cancer development (89). The link between ROS and early cancer progression fits with the timing of KRAS mutations, which first appear in PanIN1 lesions. Along these lines, a reduction in mitochondrial ROS using a mitochondrial antioxidant, mitoQ, actually thwarted PanIN formation in an animal model (86).

However, as PDA precursor lesions advance, and invasive PDA matures, the stroma becomes more pronounced, conditions are more severe, and nutrients are in shorter supply. The nutrient-deprived stroma becomes more oxidative under these conditions. More specifically, low glucose conditions drive a surge in ROS levels (50, 90–92), principally because glucose is the main substrate for multiple NADPH-generating pathways. The pentose phosphate pathway and serine biosynthesis with one-carbon metabolism are perhaps the best-studied examples (90). Chemotherapy adds to ROS levels in the PDA microenvironment, which further compounds the oxidative perils routinely faced by these malignant cells (93). Thus, as the dangers of ROS mount, adverse consequences and toxicities associated with ROS start to outstrip any pro-survival benefits favoring tumor growth. Enhanced antioxidant defense mechanisms become paramount to PDA cells for survival (94).

KRAS-driven pancreatic cancer cells bypass the oxidative phase of the pentose phosphate pathway (29). Therefore, alternative NADPH-generating pathways are likely to be important for maintenance of cellular reductive power. There are 13 different metabolic enzymes known to directly interconvert NADP^+^ to NADPH, and augment the basic reducing currency in cells. These enzymes include: ME (1, 2, and 3), IDH (1 and 2), MTHFD (1 and 2), G6PD, PGD, NNT, GLUD (1 and 2), and ALDH3A1. Reducing equivalents from NADPH maintain glutathione in its reduced form. Glutathione and NADPH collaborate to biochemically prime the remainder of the antioxidant defense system, which consists of roughly 40 enzymes including superoxide dismutases, catalases, glutathione peroxidases, thioredoxins, peroxiredoxins, and glutaredoxins (95).

Son and colleagues demonstrate that malic enzyme 1 (ME1) plays an especially key role in augmenting NADPH levels in PDA. In this non-canonical NADPH biosynthesis pathway, glutamine is converted to glutamate by GLS1 in the mitochondria. Glutamate is generated and transports out of the mitochondria into the cytosol through the malate-aspartate shuttle, where it is converted to cytosolic oxaloacetate (OAA). Mitochondrial and cytosolic aspartate transaminase (GOT2 and GOT1, respectively) are required for this sequence (57). After additional oxidative reactions in the cytosol, ME1 finally yields NADPH. As seen before, oncogenic KRAS appears to influence this adaptive PDA redox program. The authors, and others identified GOT1 and GOT2 as promising therapeutic targets based on these biologic insights (57, 96).

Oncogenic KRAS also induces the transcription of the NRF2 transcription factor (97). NRF2 positively regulates antioxidant defense elements, such as genes that drive glutathione synthesis, glutathione peroxidase, glutathione reductase, glutathione transferases, thioredoxins, and several NADPH-generating enzymes (G6PD, GPD, IDH1, and ME1) (98, 99). Additionally, NAD^+^ kinase (NADK) was recently identified as an additional component of PDA antioxidant defense. The enzyme converts NAD^+^ to NADP^+^, and is upregulated in PDA cells, as compared to normal cells. Silencing NADK increased ROS levels, and also diminished PDA growth in cell lines and *in vivo* (100).

**Figure 4:**
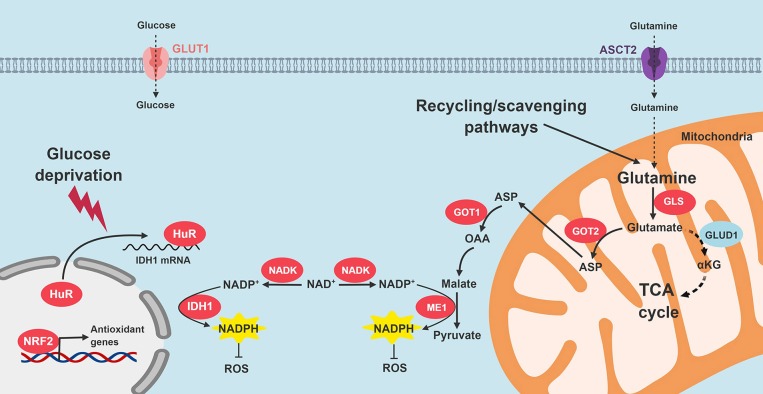
Signaling pathways that restore redox homeostasis in PDA. Nutrient scarcity in the PDA microenvironment augments oxidative stress. Multiple adaptive strategies are recruited by PDA cells. KRAS signaling promotes NADPH production through glutamine catabolism, followed by ME1 activity. In addition, NRF2 and NADK are upregulated. HuR cytoplasmic translocation stabilizes IDH to enhance reductive power. GLS, glutaminase; GOT, aspartate transaminase; ME1, malic enzyme 1; IDH1, isocitrate dehydrogenase 1; TCA, tricarboxylic acid; ROS, reactive oxygen species; NADK, NAD^+^ kinase.

We recently reported that HuR enhances antioxidant defense through post-transcriptional stabilization of the NADPH-generating enzyme, IDH1 (50). When PDA cells are exposed to an acute oxidative stress, HuR rapidly binds to the 3′-untranslated regions of IDH1 transcripts, stabilizes the transcript, increases IDH1 protein expression and activity, augments NADPH levels, and reduces intracellular ROS (50). The whole process is executed in just a few hours, which enables PDA to respond to acute oxidative stress in short order. Genetic modulation of IDH1 with siRNAs reduced PDA survival under glucose withdrawal more than any other NADPH-generating enzyme (Vaziri-Gohar, unpublished). While IDH1 mutations are oncogenic in other cancer types, our work highlights the importance of wild-type IDH1 in PDA pathogenesis. Moreover, while IDH1 is cytosolic, enhanced reductive power related to this enzyme also appears to impact redox levels in the mitochondria [Vaziri-Gohar, unpublished, and also (101, 102)]. Figure 4 summarizes PDA adaptations that restore redox balance.

## Conclusion and Summary of Metabolic Vulnerabilities in PDA

While the molecular determinants of aggressive PDA biology have not been definitively determined, it seems plausible that the adaptive mechanisms used by PDA cells to overcome the harsh metabolic milieu also contribute to the aggressive and chemotherapy-resistant phenotype responsible for poor patient outcomes. PDA's transformation toward a seemingly invincible state is akin to a runner training at high altitudes or a cactus surviving in a desert. These performance enhanced cells simply cannot be eradicated by conventional DNA targeting agents (i.e., chemotherapy) currently in use. A better understanding of the metabolic dependencies needed to survive harsh conditions will likely uncover metabolic vulnerabilities, and these alternative therapeutic strategies are not likely to be as critical to well-perfused normal cells.

In this review, we highlighted mutant KRAS as an important player in adaptive metabolic reprogramming to the PDA microenvironment. However, new targets or strategies have also come to light, including NRF2, HuR, mitochondrial biology, non-canonical nutrient acquisition processes (autophagy, macropinocytosis, alanine uptake), and antioxidant defense (NADPH-generating enzymes like ME1 and IDH1) (Table 1). There is a growing interest in exploiting new insights into cancer metabolism with good reason. A number of clinical trials targeting metabolic pathways in patients with PDA have been completed or are underway (Table 2).

**Table 1:** Metabolic dependencies in PDA.

**Event**	**Mediated by**	**References**
**MITOCHONDRIAL METABOLISM**		
Increased oxidative phosphorylation	Down regulation of Drp1	(61)
	HuR	(50)
Increased biogenesis	HuR	Unpublished
**NUTRIENT ACQUISITION**		
Autophagy	Oncogenic KRAS	(74)
NAD^+^ salvage pathway	miR-206	(78, 79)
Macropinocytosis	Oncogenic KRAS	(35)
Increased alanine uptake	Oncogenic KRAS	(85)
**REDOX HOMEOSTASIS**		
Upregulation of ME1	Oncogenic KRAS	(57)
Upregulation of NRF2	Oncogenic KRAS	(97)
Upregulation of IDH1	HuR	(50)

**Table 2:** Clinical trials targeting key steps of PDA metabolism.

**Target**	**Agent**	**Phase and status**	**NCT no**.	**References**
**MITOCHONDRIAL OXPHOS**				
ETC	Metformin + gemcitabine + ertotinib	II; Completed	NCT01210911	(63)
	Metformin + paclitaxel	II; Completed	NCT01971034	(103)
	Metformin + gemcitabine/nab-paclitaxel	I; Recruiting	NCT02336087	
	Metformin + mFOLFIRINOX	II; Active	NCT01666730	
	Metformin + rapamycin	Ib; Active	NCT02048384	
	Metformin + radiosurgery	I; Active	NCT02153450	
TCA cycle	CPI-613	I; Completed	NCT01839981	
	CPI-613 + mFOLFIRINOX	I; Active	NCT01835041	(67)
	CPI-613 + gemcitabine/nab-paclitaxel	I; Active	NCT03435289	
**NUTRIENT ACQUISITION**				
Autophagy	HCQ	II; Completed	NCT01273805	(104)
	HCQ + gemcitabine	I/II; Active	NCT01128296	
	HCQ + gemcitabine + abraxane	I/II; Active	NCT01506973	
	CQ + gemcitabine	I; Completed	NCT01777477	(105, 106)
**REDOX HOMEOSTASIS**				
Glutaminase	CB-839	I; Active	NCT02071862	

*HCQ, hydroxychloroquine; CQ, chloroquine; mFOLFIRINOX, modified FOLFIRINOX*.

## Author Contributions

AV-G and JW wrote the manuscript and prepared the figures. MZ and JB critically reviewed the manuscript. AV-G and JW approved the final manuscript.

### Conflict of Interest Statement

The authors declare that the research was conducted in the absence of any commercial or financial relationships that could be construed as a potential conflict of interest.

## Abbreviations

ALDH3A1: aldehyde dehydrogenases
ASCT2: alanine-serine-cysteine transporter 2
dCTP: deoxycytidine triphosphate
Drp1: dynamin-related protein 1
EIPA: 5-(*N*-ethyl-*N*-isopropyl)amiloride
G6PD: glucose-6-phosphate dehydrogenase
GLUD: glutamate dehydrogenase
GLUT1: glucose transporter 1
HIF-1α: hypoxia-inducible factor-1a
IDH: isocitrate dehydrogenase
LDH: lactate dehydrogenase
ME: malic enzyme
MTHFD: methylenetetrahydrofolate dehydrogenase
NAD^+^: oxidized nicotinamide adenine dinucleotide
NADK: NAD^+^ kinase
NADP^+^: oxidized nicotinamide adenine dinucleotide phosphate
NADPH: reduced nicotinamide adenine dinucleotide phosphate
NAMPT: nicotinamide phosphoribosyltransferase
NMN: nicotinamide mononucleotide
NNT: nicotinamide nucleotide transhydrogenase
NRF2: nuclear factor erythroid 2 related factor 2
OAA: oxaloacetate
OXPHOS: oxidative phosphorylation
PanIN: pancreatic intraepithelial neoplasia
PDA: pancreatic ductal adenocarcinoma
PDHK1: pyruvate dehydrogenase kinase 1
PGD: phosphogluconate dehydrogenase
PPP: pentose phosphate pathway
ROS: reactive oxygen species
shRNA: short hairpin RNA
TCA: tricarboxylic acid.
