# Corrigendum: Metabolic Dependencies in Pancreatic Cancer

**DOI:** 10.3389/fonc.2018.00672

**Published:** 2019-01-31

**Authors:** Ali Vaziri-Gohar, Mahsa Zarei, Jonathan R. Brody, Jordan M. Winter

**Affiliations:** ^1^School of Medicine, Case Western Reserve University, Cleveland, OH, United States; ^2^Department of Veterinary Physiology and Pharmacology, Texas A&M University, College Station, TX, United States; ^3^Department of Medicine, Brigham and Women's Hospital and Harvard Medical School, Boston, MA, United States; ^4^Division of Surgical Research, Department of Surgery, Jefferson Pancreas, Biliary and Related Cancer Center, Jefferson Medical College, Thomas Jefferson University, Philadelphia, PA, United States; ^5^Department of Surgery and Division of Surgical Oncology, University Hospitals Cleveland Medical Center, Cleveland, OH, United States

**Keywords:** pancreatic cancer, metabolism, redox homeostasis, metabolic dependencies, targeting metabolism

In the original article, all references for in Tables 1, 2 were incorrectly listed. The corrected references for both Table 1 and Table 2 have been corrected and provided below.

**Table 1:** Metabolic dependencies in PDA.

**Event**	**Mediated by**	**References**
**MITOCHONDRIAL METABOLISM**		
Increased oxidative phosphorylation	Down regulation of Drp1	(61)
	HuR	(50)
Increased biogenesis	HuR	Unpublished
**NUTRIENT ACQUISITION**		
Autophagy	Oncogenic KRAS	(74)
NAD^+^ salvage pathway	miR-206	(78, 79)
Macropinocytosis	Oncogenic KRAS	(35)
Increased alanine uptake	Oncogenic KRAS	(85)
**REDOX HOMEOSTASIS**		
Upregulation of ME1	Oncogenic KRAS	(57)
Upregulation of NRF2	Oncogenic KRAS	(97)
Upregulation of IDH1	HuR	(50)

**Table 2:** Clinical trials targeting key steps of PDA metabolism.

**Target**	**Agent**	**Phase and status**	**NCT No**.	**References**
**MITOCHONDRIAL OXPHOS**				
ETC	Metformin + gemcitabine + ertotinib	II; Completed	NCT01210911	(63)
	Metformin + paclitaxel	II; Completed	NCT01971034	(103)
	Metformin + gemcitabine/nab-paclitaxel	I; Recruiting	NCT02336087
	Metformin + mFOLFIRINOX	II; Active	NCT01666730
	Metformin + rapamycin	Ib; Active	NCT02048384
	Metformin + radiosurgery	I; Active	NCT02153450
TCA cycle	CPI-613	I; Completed	NCT01839981	
	CPI-613 + mFOLFIRINOX	I; Active	NCT01835041	(67)
	CPI-613 + gemcitabine/nab-paclitaxel	I; Active	NCT03435289
**NUTRIENT ACQUISITION**				
Autophagy	HCQ	II; Completed	NCT01273805	(104)
	HCQ + gemcitabine	I/II; Active	NCT01128296	
	HCQ + gemcitabine + abraxane	I/II; Active	NCT01506973
	CQ + gemcitabine	I; Completed	NCT01777477	(105, 106)
**REDOX HOMEOSTASIS**				
Glutaminase	CB-839	I; Active	NCT02071862

*HCQ, hydroxychloroquine; CQ, chloroquine; mFOLFIRINOX, modified FOLFIRINOX*.

In the original article, references in Table 2 were not provided in the reference list. The references have now been inserted.

The authors apologize for these errors and state that they do not change the scientific conclusions of the article in any way. The original article has been updated.
